# A Call for Broadening the Analysis of Corporate Political Activities: Insights From Social Media as a Commercial Determinant of Health: Comment on "Corporate Political Activity: Taxonomies and Model of Corporate Influence on Public Policy"

**DOI:** 10.34172/ijhpm.9155

**Published:** 2025-09-22

**Authors:** Teresa Leão

**Affiliations:** ^1^EPIUnit ITR, Institute of Public Health of the University Porto, University of Porto, Porto, Portugal.; ^2^Departamento de Ciências da Saúde Pública e Forenses, e Educação Médica, Faculdade de Medicina, Universidade do Porto, Porto, Portugal.

**Keywords:** Commercial Determinants Health, Unhealthy Commodity Industries, Social Media, Misinformation, Disinformation, Corporate Social Responsibility

## Abstract

Corporate political activities of unhealthy commodity industries have been identified and categorised in terms of *framing* and *action* strategies. This commentary discusses the relevance of systematically and comprehensively applying these taxonomy models to other commercial determinants of health, with special attention to their joint activities and health implications. The social media industry is an especially relevant case to be analysed due to its direct and indirect health effects, namely those associated with digital marketing of unhealthy commodities, mis and disinformation, and social polarisation. Interferences in research, lobbying, and corporate social responsibility actions are examples of the strategies used by this industry to prevent and obstruct regulators’ efforts, limiting the control of the marketing of unhealthy products, the spread of mis/disinformation, and the promotion of violent speech and attitudes.

## Introduction

 It is widely recognised that several industries directly influence populations’ health through their products and services.^1^ Indeed, some corporations and alliances follow business models and strategies to promote their economic growth, which can, positively or negatively, influence individuals’ behaviours, health status, and health equity.^2^

 Globalisation, with increasing market coverage, widespread supply chain and marketing of unhealthy products and services in high-, middle-, and low-income countries, has been accelerating these detrimental effects.^2^ In 2024, the World Health Organization (WHO) alerted that four industries—tobacco, alcohol, ultra-processed foods, and fossil fuels—were responsible for the loss of 19 million lives per year worldwide.^1^ These are considered unhealthy commodity industries, as they include unhealthy products or services as part of their portfolios and reach a large number of consumers, contributing to these industries’ profits but also to premature mortality and morbidity.^1^

 Focusing on these industries, Ulucanlar et al^3^ proposed a model of taxonomies to support the analysis of their corporate political activity. Two taxonomies – on *framing* and *action* strategies – allow the systematic analysis of the influence posed by tobacco, alcohol, ultra-processed foods and beverages, and gambling. Yet, other industries with positive or negative, direct or indirect, health effects driven by their services, practices, and pathways, can also be considered commercial determinants of health,^4^ such as pharmaceuticals, insurance, transportation, or social media corporations.^4^ This commentary aims to further develop the discussion on how (1) these taxonomy models may contribute to identifying corporate political activity from other commercial determinants of health beyond the unhealthy commodity industries, and (2) these analyses can unveil the risks of the joint action of different commercial determinants of health. The case of social media will be used to introduce these perspectives.

## Commercial Determinants of Health: The Case of Social Media

###  Direct and Indirect Health Effects 

 Public health research on the health impacts of unhealthy commodity industries is extensive. Yet, other industries can also impact health through the services and products they make available, disseminate, and publicise, even if unhealthy commodities are not their main products.^4^ Broadening the analysis of corporate political activities to other commercial determinants of health may be relevant to informing public health actors and policy-makers, and preventing policy interferences.

 Social media is one example of a commercial determinant of health,^5^ with a potentially positive role in promoting healthy behaviours and social connection, but also in unhealthy products’ marketing, mis/disinformation about health technologies, and on users’ psychological health. Created as a form of mass media communication, where information and personal messages are shared online by users, it functions not only as a product, used by these individuals to create, share, and access content, but also as a tool for institutions and organizations to reach a broad number of (potential) customers. Its health impacts – positive and negative – follow different pathways.

 Firstly, social media allows unlimited access to information from trustworthy media channels or organizations (such as the Centers for Disease Control and Prevention or the WHO), linked with the possibility of engaging directly with health experts or professionals, potentially contributing to promote health literacy and healthy behaviours. However, in recent years, alerts were launched about the risks of rapid dissemination of mis/disinformation—unintentionally or deliberately created and disseminated—on vaccines and control of communicable (such as COVID-19) and non-communicable diseases and disorders (such as cancer or autism).^6^

 Secondly, users can instantly publish content, regardless of their expertise on the topic or the accuracy of the information, and publications can be rapidly disseminated, reaching large numbers of people.^6^ As these platforms’ profit directly depends on users’ engagement and content sponsors, and since emotional, subversive, and divisive publications have a higher likelihood of engagement and dissemination, the financial incentive to maintain mis/disinformation, frequently allied to emotional content, exists, independently of its potential negative effects on health.^6^

 Thirdly, as this industry fosters the connection between people with common interests, it has the potential to maintain social connections, improve social capital, and contribute to users’ well-being.^6^ However, it has been shown that passive engagement is not as beneficial as active engagement, and the excessive use of these platforms can increase feelings of loneliness.^6^ Its addictiveness, through features of infinite scrolling and immediate reward, can trigger social comparison, loss of self-esteem, and obsessive-compulsive behaviours.^6^ Furthermore, as these networks tend to approximate persons with similarities, they promote *echo chambers* where social polarisation, mistrust, and reinforcement of hate speech may emerge.^6^

 Moreover, algorithms contribute to the effective dissemination of information and addictive features of these platforms. Besides the automatic creation of content, the identification of target groups, based on users’ gender, age, geography, social media data, content viewed, and responses given (or not) to it, is extensively used to effectively obtain engagement, promote visualizations, and resharing. Additionally, bots are used to automatically and autonomously publish and share content, react and message, and some of these bots can create and disseminate mis/disinformation, often on health and politics, using formulas to promote higher engagement, sharing, and re-sharing.^6^

 As such, with the use of these strategies and the economic interests in their maintenance, mis/disinformation, polarisation, and violent speech have become more common, fostering fear, anxiety, and jeopardizing trust in institutions and evidence-based health information.^6^ Antivaxx movements and, subsequently, measles outbreaks are one of its consequences.

###  The Interaction Between Commercial Determinants of Health

 Cooperation between industries can increase their influence on social and political domains.^3^ Joint strategic investments, ownership of companies, or crossed affiliations as, for example, participation in directors’ boards of other companies, may promote similarities and synergies in their management.^7^ Common public relations, marketing firms, legal consultants, and lobbyists’ contracts create a blueprint for lobbying and marketing strategies across sectors.^7^

 Links between social media and the other diverse commercial determinants of health are increasingly evident, with the WHO warning about the use of social media by the tobacco,food, and alcohol industries to target younger generations.^8^ In 2020, it was predicted that, by 2025, digital ads on alcohol would reach over 600 billion US dollars, accounting for more than half of the total ad expenditure.^8^ The tobacco industry has been promoting new tobacco products on social media, reaching over 385 million people, of whom 16 million were minors.^9^ Since the functioning of social media platforms is dependent on engagement, sponsoring, and advertising, the marketing of these substances on these platforms is welcome, despite their health risks. As such, unverified, potentially harmful content can be disseminated using algorithms, bots, and content creators, and, according to the WHO, target minors.^8,10^

 Content creators also come to play a central role in this interaction. They not only disseminate information and attitudes but also engage in the marketing of unhealthy products. Since their profit depends on their followers’ engagement and content sponsoring, they create emotive, novel, or controversial information to engage response, visualisations and followers. This attracts brands to market their products through these actors, with financial benefit for the three parties (content creators, social media platforms, and other industries).^6^ Content creators are thus capitalized by the alcohol, tobacco, and food industries to promote their products on social media, including among younger groups.^10,11^

## Regulation and Policy Influence: Can Corporate Political Activities’ Taxonomies Be Applied to Social Media?

 Regulating social media would allow for the regulation of marketing practices by the tobacco, alcohol, and ultra-processed foods industries, among other commercial determinants of health. As such, several organisations have been calling on regulators to act, with the WHO publishing protocols to facilitate the monitoring of the marketing of unhealthy products to minors, in the media, the internet, and social media,^10^ and guidance on restrictions on digital marketing, including through social media.^11^ The regulation of marketing strategies involving social media can be more challenging than in traditional media in terms of jurisdiction to legislate, adjudicate, and enforce,^11^ but diverse countries have developed efforts, from comprehensive to partial restriction strategies focused on specific product categories, vulnerable groups, or on data collection, processing, and use.^11^

 Like other industries, social media platforms may try to interfere with regulatory processes through diverse strategies. Thus, the two taxonomies proposed by Ulucanlar et al^3^ – on *framing* and *acting* – may be useful in informing the strategies used by this industry. Some of those have been previously identified,^5,6,12^ but not framed as corporate political activities.

 Social media platforms try to influence how they, their policies, and regulators’ proposals are perceived by policy-makers, the public, and civil society, and persuade of the legitimacy or importance of their actions. One example of these strategies is corporate social responsibility initiatives,^3^ such as the Free Basics programme, launched by Meta to provide internet access to deprived areas, reaching 28 African countries by 2019.^12^ This platform was blamed for “digital experiments and data extraction” in disadvantaged, unregulated settings, collecting large data sets and data streams with metadata from all user activities, which could be used to train algorithms, and create, test, and promote products.^12^ Philanthropic initiatives like this try to place these industries as relevant economic and social actors, framing them as *good *actors and socially responsible, nurturing their positive reputation, and creating public support.^3^

 Another example is the collaboration between these platforms and health organisations on correcting mis/disinformation.^12^ During the COVID-19 crisis, Meta has promised to invest in eliminating harmful content and disseminating evidence-based information.^12^ This effort—paradoxical and ineffective since it does not prevent mis/disinformation practices—can reinforce its framing as a *good* actor, responsible and respectable, capable of understanding the urgency to tackle these risks.^3^ Besides contributing to a favourable framing, these collaborations create alliances with key organizations, influencing not only public support but also experts’ and policy-makers’ favourable views, managing its reputation to ensure corporate advantage and guaranteeing a place in policy-making processes.^3^

 Furthermore, alliances and corporate social responsibility initiatives can support platforms’ discourse around their interest in promoting users’ well-being and safety, and compromise in self-regulation.^3^ Despite being a common discourse, it has been shifting directions over time, adapting to social and political contexts.^13^ After years of lobbying against regulations,^13^ in early 2020, Meta asked for more regulation on sponsored political content, in response to a *tech lash*.^14^ In 2020 and 2021, it invested in self-regulation efforts, hiring external fact-checking services, removing content, and adding warnings.^12^ Contrarywise, in 2025, it announced that this third-party triage would no longer exist, holding the users responsible for deciding which publications must be considered mis(dis)information.^15^ This recent position aligned with the free speech argument, which is frequently used to frame regulations as limiting individuals’ basic rights.^3^

 Research funding and control have also been identified by Zenone et al in their 2023 viewpoint.^12^ Social media platforms have, on the one hand, funded studies on misinformation, but on the other, in 2021, misinformation researchers, analysing political advertising content, saw their accounts removed.^12^ Strict barriers exist for researchers to access data (namely key metrics), and data scraping is not allowed, limiting the transparent monitoring of trends and the impact of social media use.^12^

 An analysis of these corporations’ practices can contribute to better understand how they influence regulatory processes. This brief has unveiled some of the actions and discourses used by social media corporations, and the pivotal interaction between this industry and other commercial determinants of health. However, a systematic and comprehensive analysis of their corporate political activities remains essential to inform policy-making and effectively support regulations of digital marketing, preventing its negative health impacts. This is especially relevant since social media platforms not only disseminate information and influence health behaviours but also seem to have a close relationship with some decision-makers, with revolving door practices jumping into the news headlines after the 2024 US political elections.^16^ Thus, these taxonomies^3^ must be used to monitor the corporate political activities of these corporations and their interaction with other commercial determinants of health, with special attention to the interaction with the political sphere.

## Ethical issues

 Not applicable.

## Conflicts of interest

 Author declares that she has no conflicts of interest.
